# Automatic Identification of First-Order Veins and Corolla Contours in Three-Dimensional Floral Images

**DOI:** 10.3389/fpls.2020.549699

**Published:** 2020-09-16

**Authors:** Yi-Hsiang Wang, Hao-Chun Hsu, Wen-Chieh Chou, Chia-Hao Liang, Yan-Fu Kuo

**Affiliations:** Department of Biomechatronics Engineering, National Taiwan University, Taipei, Taiwan

**Keywords:** three-dimensional image, floral traits, corolla contours, first-order veins, pollination type, Gesneriaceae, Ligeriinae, venation

## Abstract

Defining and quantifying corolla traits are essential for studying corolla shape variation. Three-dimensional (3D) images of corollas contain comprehensive information regarding corolla structures and are optimal for studying corolla shapes. Conventionally, corolla traits are identified and quantified manually from 3D images. Manual identification is time consuming and labor intensive. In this study, approaches are proposed to automatically identify first-order veins and corolla contours in 3D corolla images. The first-order veins of the corollas were identified using Hessian of Gaussian and Dijkstra’s algorithm. The contours of the corollas were identified using vector harmony and node distance thresholding. A total of 130 3D images of 28 species in the subtribe Ligeriinae were collected and used to test the proposed approaches. The successful detection rate reached 86.54%. Two derived traits, contour–vein ratio and corolla angle, were defined and quantified using the first-order veins and corolla contour results to investigate the relationship between corolla shapes and pollination types of the subtribe Ligeriinae. Analyses revealed that the mean corolla contour, mean absolute corolla angle, and mean contour–vein ratio of the ornithophilic species were significantly smaller compared with the other species. The mean corolla contour, mean corolla angle, and mean contour–vein ratio of the melittophilic species were significantly larger compared with those of the ornithophilic species. The proposed method was also applied to certain Gesneriaceae species in the subtribes Gloxiniinae, Streptocarpinae, and Didymocarpinae. The results revealed that the method could be applied to most fresh sympetalous flowers for identifying first-order veins and corolla contours.

## Introduction

The biology of corollas, essential organs for most angiosperm species, has attracted considerable research attention. Corollas are three-dimensional (3D) objects with complex shapes and sizes. Authentic 3D images of corolla are required to capture the complexity of their structural information. Since the early 21st century, as a result of advancements in 3D imaging, nondestructive investigation of plant materials has become feasible and affordable (Stuppy et al., 2003; Karahara et al., 2015; Mathers et al., 2018; Hesse et al., 2019). Structural information captured in 3D images has been further combined with geometric and morphometric techniques to assess the variations in corolla shape and size (van der Niet et al., 2010; Wang et al., 2015). This technical integration allows for a precise and objective investigation of corollas.

A few studies have employed 3D technologies to examine the morphological properties of corollas. Hsu et al. (2017) examined the association of 3D petal traits and the genotypes in a hybrid line of *Sinningia* cultivars. Reich et al. (2019) identified the modularity and evolution of 3D flower shapes in *Erica* species. Yao et al. (2019) evaluated the morphological type of 3D petal shapes in *Nigella* species. Hsu et al. (2020) identified a series of 3D corolla traits that had strong associations with pollination types and contained significant phylogenetic signals in *Corytholoma* species. These studies successfully assessed variations in the morphological properties of corollas. However, most of the procedures for quantifying morphological properties in the proposed approaches rely heavily on manual operations. As well as being time consuming and labor intensive, such manual measurements may have different criteria among researchers, and criteria may be inconsistently followed because of fatigue. Therefore, approaches for automatically and objectively measuring the morphological properties of corollas would be valuable.

Researchers of several medical studies have proposed approaches for automatically identifying human tissue and organs in 3D images. Lidayová et al. (2017) identified the skeleton of the vein system by characterizing the voxel intensities between vein tissue and other tissues. Montúfar et al. (2018) isolated the contour of the craniofacial structure by mapping sectional slice images to a 3D volumetric image. Idram et al. (2019) positioned anatomical points by computing the geometric properties of a 3D surface image. The proposed approaches in each study were developed to achieve specific objectives using various techniques. Directly applying a medical imaging strategy to research materials other than human tissue is a challenge. Therefore, in this study, we propose approaches designed specifically for identifying corolla traits from 3D images acquired using micro-computed tomography (micro-CT).

**Figure 1:**
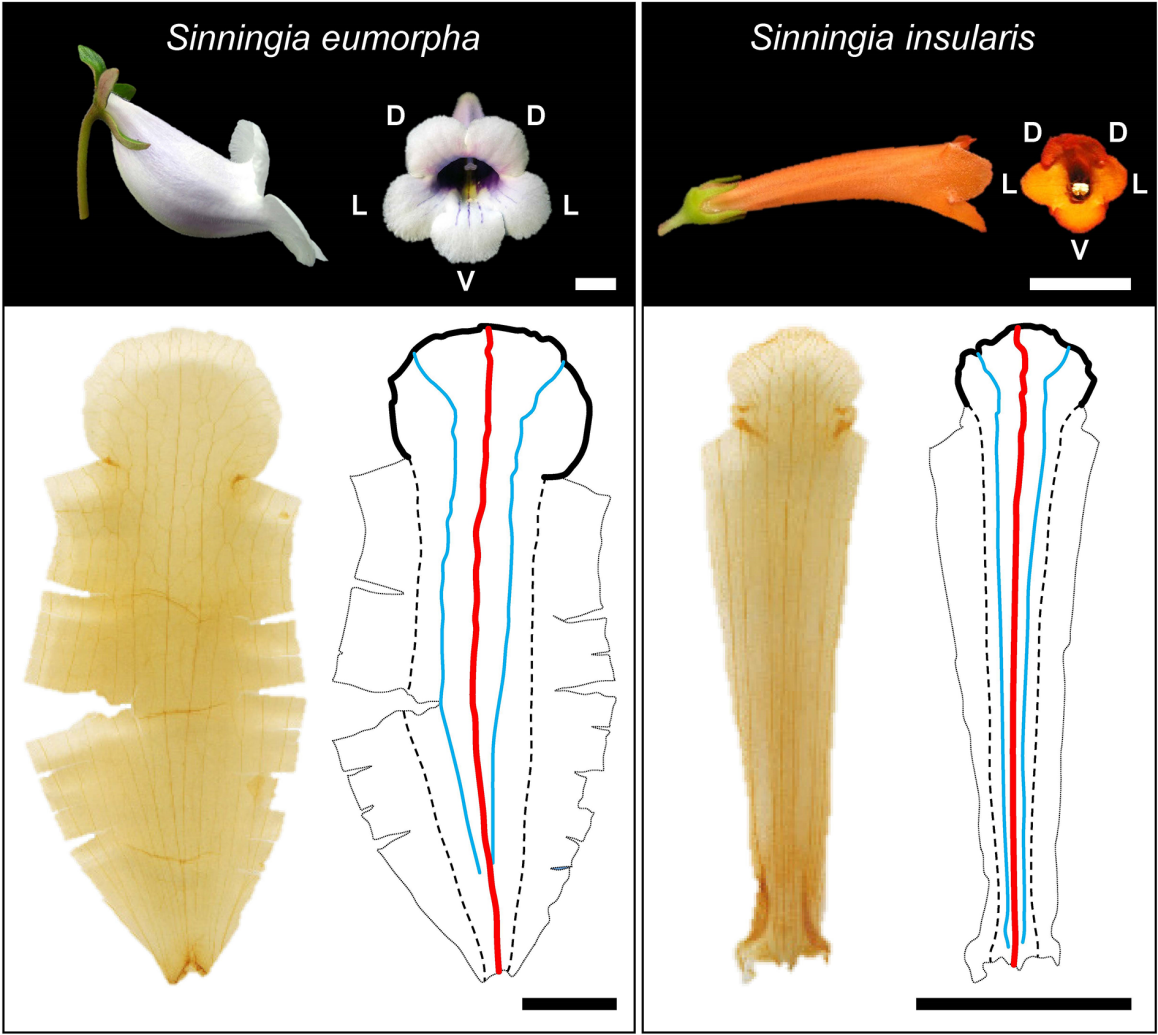
Corolla photographs, images of ventral petals after tissue clearing treatment, and illustrations of veins and corolla contours. D, L, and V denote dorsal, lateral, and ventral positions, respectively. Black solid line: corolla contour; red solid line: first-order vein; blue solid line: second-order vein; dashed line: contour of the tube–tube connected rim. Scale bar: 1 cm.

Corollas from species in the subtribe Ligeriinae are excellent materials for developing approaches for identifying corolla traits using 3D images acquired from micro-CT. The subtribe Ligeriinae is a monophyletic group that yields flowers with considerable variations in size (1–9 cm in length) and shape (tubular-, funnel-, and bell-shaped). The rapid change of corolla morphology in a monophyletic group also provides the opportunity to study the association between corolla shape and pollinators and the evolutionary and developmental biology of corollas (Perret et al., 2007). The corollas in this subtribe typically have a five-petal structure. Each petal contains a venation of a prominent first-order vein in the middle of a petal (red lines in **Figure 1**) and a contour of the lobe edge, also referred to as a lobe contour (black lines in **Figure 1**). The veins and contours of petals were captured in a 3D volumetric image and a 3D surface image through micro-CT imaging (Wang et al., 2015). To investigate the geometric properties of corollas, an approach that automatically identifies vein skeletons and corolla contours in 3D images was developed.

The objectives of this study were to 1) establish algorithms for automatically detecting the first-order veins and corolla contours in 3D images of Ligeriinae corolla specimens, 2) define and quantify corolla angle and contour–vein ratio, 3) examine the association between the two quantified traits and pollination types, and 4) verify the applicability of the proposed algorithms to the species in the family Gesneriaceae other than the subtribe Ligeriinae.

## Materials and Methods

### Flower Material

The plants of 28 species in the subtribe Ligeriinae (**Supplementary Table 1**) were provided by Cecilia Koo of the Botanic Conservation Center, Pingtung, Taiwan. All plants were cultivated in a greenhouse under natural lighting, 70%–80% humidity, and a temperature of 22–28°C (Technology Commons X, College of Life Science, National Taiwan University, Taiwan). For each species, three to 14 floral specimens were collected, for a total of 130 specimens. The acquired specimens were freshly collected.

### Raw Image Acquisition

**Figure 2:**
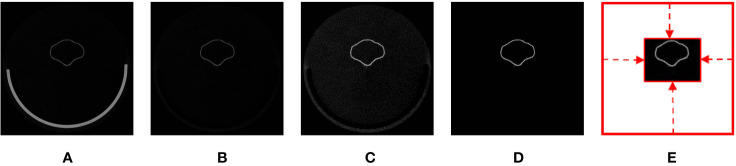
Illustration of volumetric image generation: **(A)** original image, **(B)** base-eliminated image, **(C)** contrast-adjusted image, **(D)** noise-removed image, and **(E)** void space–removed image.

Three-dimensional raw images of the specimens were acquired using a micro-CT scanner (SkyScan 1076, Bruker; Kontich, Belgium). The fresh specimens were cut from petioles at full bloom and immediately fixed to the base of the inside of the scanner chamber. The scanning procedures and parameters followed those of Wang et al. (2015). After a scan was performed, a 3D raw image of the specimen was created using a SkyScan NRecon software (Bruker; Kontich, Belgium). The 3D raw images were composed of hundreds of 2D slices in grayscale (**Figure 2A**). The nominal resolution of the images was 36.54 μm in each dimension. In terms of dimension, depending on the size of a specimen, the slices were 1000 × 1000 pixels or 1968 × 1968 pixels (**Supplementary Table 1**). The raw images with slices of 1968 × 1968 pixels were downsized to 50% in each dimension to reduce memory requirements and computation complexity. The file sizes of the raw images ranged between 0.5 and 1.7 GB.

### Volumetric Image Generation

Volumetric images that described the textural properties of the corollas were generated from raw images. During volumetric image generation, algorithms were applied to the raw images to eliminate the base of the scanner, adjust image contrast, reduce noise, and remove regions of noninterest. Base elimination and contrast adjustment were performed following the procedure of Wang et al. (2015) (**Figures 2B, C** **)**. To reduce noise and remove regions of noninterest, a contrast-adjusted image (**Figure 2C**) was converted to a binary image using a threshold value of zero. 3D connected-component labeling (CCL, He et al., 2017) using 26-voxel connectivity was applied to the binary image. The object with the maximum volume in the binary image was regarded as the corolla. The rest of the isolated voxels were considered noise (i.e., sparkles) and were removed. The raw image was then masked (Lien et al., 2013) using the binary image to retain the corolla (**Figure 2D**). The void space surrounding the corolla in the raw image was identified and eliminated to reduce the size of the image (**Figure 2E**). The resultant image was referred to as a volumetric image. The file sizes of the volumetric images ranged from 0.05 to 0.14 GB. The aforementioned operations were perform using MATLAB (The Mathworks; Natick, MA, USA; see **Supplement Data 1** for the MATLAB script).

### Surface Image Generation

Surface images were generated from the volumetric images to describe the shapes of the corollas. A surface image was composed of nodes covering the surface of a corolla. In the volumetric images, the corollas had high grayscale values, whereas the void space had a grayscale value of 0. To generate a surface image, voxels with grayscale values of 10 in a volumetric image were first identified as the nodes of the corolla. The density of the nodes was then reduced to 1% of the original density using surface simplification (Pauly et al., 2002). The collection of the nodes was referred to as a surface image (see **Supplement Data 1** for the MATLAB script).

### Identification of First-Order Veins

#### Detection of Venation

**Figure 3:**
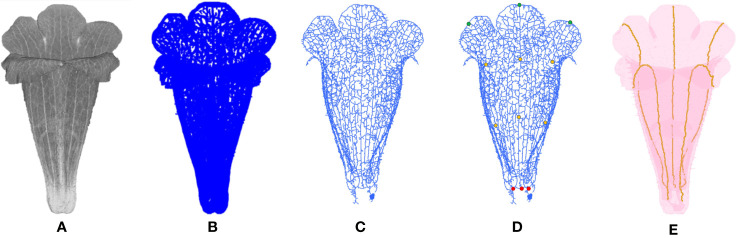
Illustration of venation detection on a corolla of *S. conspicua*: **(A)** volumetric image, **(B)** initial venation, **(C)** skeletonized venation, **(D)** start and end points of the first-order veins, and **(E)** first-order veins. The green, orange, and red points in **(D)** are distal, middle, and proximal points, respectively. For **(C, D)**, the detected venation of the dorsal petals was removed for a clear illustration.

The venation, known as the architecture of veins, of the corollas was identified from the volumetric images. The density of venation was typically higher compared with the density of neighboring mesophyll tissues. Therefore, the voxels of venation in the volumetric images were associated with larger grayscale values (**Figure 3A**). The grayscale differences between venation and mesophyll tissues werecharacterized using second derivatives (Hessian; Takeda et al., 2006). Let $I\left(x,y,z\right)\in {\mathbb{R}}^{3\times 3\times 3}$ represent the grayscale value of a voxel (*x, y, z*) and its 26 adjacent voxels. The Hessian of Gaussian ***H***(*x, y, z, σ*) (Lefkimmiatis et al., 2012) at the voxel was expressed as

(1) $$H\left(x,y,z,\sigma \right)=\sigma^{\lambda }\left[\begin{array}{ccc} I*\frac{\partial^{2}G}{\partial^{2}x} & I*\frac{\partial^{2}G}{\partial x\partial y} & I*\frac{\partial^{2}G}{\partial x\partial z}\\ I*\frac{\partial^{2}G}{\partial y\partial x} & I*\frac{\partial^{2}G}{\partial^{2}y} & I*\frac{\partial^{2}G}{\partial y\partial z}\\ I*\frac{\partial^{2}G}{\partial z\partial x} & I*\frac{\partial^{2}G}{\partial z\partial y} & I*\frac{\partial^{2}G}{\partial^{2}z} \end{array}\right]\in {\mathbb{R}}^{3\times 3},$$

where *G* is 3D Gaussian function; therefore,

(2) $$G\left(x,y,z,\sigma \right)=\frac{1}{{\left(\sigma \sqrt{2\pi }\right)}^{3}}\cdot e^{\frac{-(x^{2}+y^{2}+z^{2})}{2\sigma^{2}}}\in \mathbb{R},$$

where *σ* is the variance of the Gaussian function, and the symbol * is a convolution operation. Various *σ* values were used to identify veins of different cross-section diameters. Cross-section diameters of venation typically range from 35 to 175 μm in corollas; therefore, *σ* values of 1, 3, and 5 were used.

Suppose that the eigenvalues of ***H*** at a voxel (*x, y, z*) were *λ*_1_(*σ*), *λ*_2_(*σ*), and *λ*_3_(*σ*) in descending order (i.e., *λ*_1_ *> λ*_2_ > *λ*_3_). The likelihood p∈ℝ that the voxel (*x, y, z*) is a venation voxel with a cross-section diameter corresponding to *σ* is expressed as follows (Frangi et al., 1998):

(3) $$p(x,y,z,\sigma )=\{\begin{array}{cc} 0 & \lambda_{2}(\sigma )>0\vee \lambda_{3}(\sigma )>0\\ \left(1-e^{\frac{R_{A}^{2}(\sigma )}{2a^{2}}}\right)\cdot e^{\frac{R_{B}^{2}(\sigma )}{2b^{2}}}\cdot \left(1-e^{\frac{S^{2}}{2c^{2}}}\right) & otherwise \end{array},$$

where *R_A_*(*σ)* is $\left|\lambda_{2}(\sigma )\right|/\left|\lambda_{3}(\sigma )\right|$, *R_B_*(*σ)* is $\left|\lambda_{2}(\sigma )\right|/\sqrt{\left|\lambda_{2}(\sigma )\lambda_{3}(\sigma )\right|}$, *S* is the Frobenius norm of ***H***, and *a*, *b*, and *c* are constants. The constants *a*, *b*, and *c* were set to 0.5, 0.5, and 500, respectively. After the cross-section diameters corresponding to the 3 *σ* values were assessed, the final likelihood that the voxel (*x, y, z*) was a venation point can be expressed as follows:

(4) $$p\left(x,y,z\right)=\underset{\sigma =1,3,5}{\max}p(x,y,z,\sigma ).$$

The aforementioned operations were applied to all the voxels in a volumetric image. The collection of the voxels with *p* values ranked in the top 30% of the corollas were regarded as representing initial venation (**Figure 3B**). The remaining voxels were ignored.

The initial venation of a corolla contained noise (e.g., fuzz on petal surfaces) and fractal veins (**Figure 3B**). Three-dimensional image processing algorithms were applied to mitigate the defects. First, 3D CCL was applied to the voxels of the initial venation. The components with volumes ranked in the bottom 5% were recognized as noise. Morphological opening and dilation (Vincent, 1993) were then applied to restore the fractured veins and reconnect the isolated veins. The structuring element for the opening and dilation was a cube with a width of 2 voxels. The results were then skeletonized (Lee et al., 1994) and determined as venation (**Figure 3C**, see **Supplement Data 1** for the MATLAB script).

#### Identification of First-Order Veins

The first-order veins of the corollas were identified (**Figure 3C**) semiautomatically. The distal and proximal points of first-order veins were selected manually using a graphic user interface developed in MATLAB. The first-order veins were then automatically identified as the shortest path between the points using Dijkstra’s algorithm (Dijkstra, 1959). Certain species had a curved urceolate structure at the tube (e.g., *S. barbata*, *S. conspicua*, *S. eumorpha*, and *S. speciosa*; **Figure 1**). Additional middle points of each first-order vein were selected to identify the first-order veins (**Figure 3D**). Dijkstra’s algorithm was applied to identify the first-order veins between the distal and middle points and between the proximal and middle points, respectively. The results were then combined to form a complete picture of first-order veins (**Figure 3E**, see **Supplement Data 1** for the MATLAB script).

### Identification of Corolla Contour

#### Detection of Initial Corolla Contour

**Figure 4:**
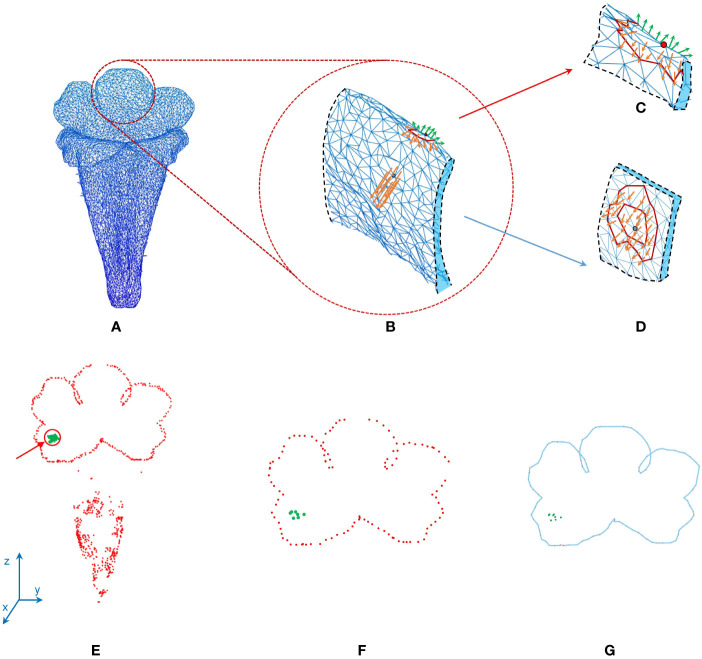
Illustration of corolla contour identification on a corolla of *S. conspicua*: **(A)** surface image, **(B)** surface image at the petal distal, **(C)** normal vectors of meshes at the corolla contour, **(D)** normal vectors of meshes in a smooth surface, **(E)** nodes with harmony smaller than 0.5, **(F)** initial corolla contour with isolated nodes (in green), and **(G)** corolla contour.

Corolla contour, defined as the edge of the corolla lobe, was identified from the surface images (**Figure 4A**). In the surface images of the corollas, three neighboring nodes were connected to form a triangular mesh (**Figure 4B**). The meshes at the corolla contour were associated with normal vectors with various directions (**Figure 4C**), whereas the meshes at the smooth surfaces of the corolla were associated with normal vectors with similar directions (**Figure 4D**). Therefore, contour nodes were identified by examing the normal vectors of the meshes surrounding the nodes.

Suppose that a triangular mesh is composed of three nodes ***x*** *_i_*, ***x*** *_j_*, and $x_{k}\in {\mathbb{R}}^{3}$. The unit normal vector ***v*** of the triangular mesh is represented as follows:

(5) $$v=\frac{\vec{x_{i}x_{j}}\times \vec{x_{i}x_{k}}}{\left|\vec{x_{i}x_{j}}\times \vec{x_{i}x_{k}}\right|}\in {\mathbb{R}}^{3},$$

where the symbol × is cross product operation. Let harmony *h* of a node be the mean absolute sum of the unit normal vectors of the meshes surrounding the node:

(6) $$h=\frac{\left|\sum_{i=1}^{n}v_{i}\right|}{n}\in \mathbb{R},$$

where *n* is the number of the triangular meshes in the 2-ring surrounding the node (e.g., the meshes surrounding the blue node in **Figure 4D**). The nodes with *h* values ≤ 0.5 were collected. The collection contained nodes at pistils, anthers, or trichomes that were falsely recognized as contour nodes (**Figure 4E**). To remove the falsely recognized nodes in these areas, the long axis of the corolla tube was identified and aligned to the z-axis in the 3D image space. A threshold was then set on the z-axis. The nodes beyond the threshold were regarded as falsely recognized nodes and were removed. The collection of the remaining nodes was regarded as an initial corolla contour (**Figure 4F**, see **Supplement Data 1** for the MATLAB script).

#### Removal of Isolated Nodes and Contour Smoothing

The initial corolla contour contained nodes that were falsely recognized as part of the contour (e.g., green nodes in **Figure 4E**). These nodes were isolated from the corolla contour and referred to as isolated nodes. Algorithms were employed to identify and remove the isolated nodes based on the distance between nodes. Suppose that there existed *m* contour nodes. The distances from a node ***x*** *_i_* to all the other nodes were represented as a vector $d_{i}\in {\mathbb{R}}^{m-1}$. A distance threshold *g* was then calculated as the weighted mean minimum distance of all the contour nodes:

(7) $$g=\frac{\sum_{i=1}^{m}\min\left(d_{i}\right)}{m}\cdot w_{j}\in \mathbb{R},$$

where *w_j_* is the weight for different iterations, *j* = 1 or 2 were employed. A thresholding operation was then applied to all the initial contour nodes. A node with a minimum distance min(***d*** *_i_*) larger than *g* was regarded as an isolated node and was removed from the initial contour. The thresholding was performed twice. The second thresholding operation was performed after smoothing, as described in the following paragraph. The weights *w*_1_ and *w*_2_ were set to 1.9 and 4, respectively.

The corolla contour was smoothed (**Figure 4F**) to differentiate between contour nodes and missing isolated nodes. Let ***x***_0_ be the node to be smoothed. Suppose there existed *q* nodes in the neighborhood of ***x***_0_. Smoothing was performed by replacing the coordinate of node ***x***_0_ using the mean coordinate of the nodes in the neighborhood:

(8) $${x^{\prime }}_{0}=\frac{x_{0}+\sum_{i=1}^{q}x_{i}}{1+q}\in {\mathbb{R}}^{3}\;,$$

where ***x*** *_i_* is the coordinate of a neighborhood node and ${x^{\prime }}_{0}\in {\mathbb{R}}^{3}$ is the new coordinate of node ***x***_0_. The neighborhood of ***x***_0_ was defined as a sphere with a radius of 10 voxels (0.3654 mm) centered at ***x***_0_. The smoothing was performed for 10 iterations. The second thresholding operation (Eq. 7) was then performed to remove the isolated nodes after smoothing. The results were regarded as the corolla contour (**Figure 4G**, see **Supplement Data 1** for the MATLAB script).

### Morphological Traits

**Figure 5:**
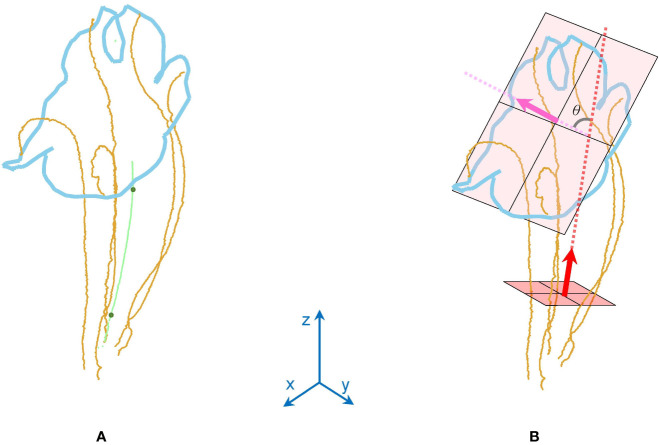
Illustration of **(A)** contour–vein ratio *ρ* and **(B)** corolla angle *θ* for a corolla of *S. conspicua*.

Two morphological traits of the corollas, contour–vein ratio *ρ* and corolla angle *θ*, were defined to examine the shape variations of the corollas. The contour–vein ratio was defined as the length of the corolla contour (blue line in **Figure 5A**) divided by the summation of the length of the five first-order veins (orange lines in **Figure 5A**). Corolla angle was defined as the angle between the lobe opening vector (pink arrow in **Figure 5B**) and the corolla central vector (red arrow in **Figure 5B**). The lobe opening vector was calculated as the normal vector of the plane that optimally fits the nodes at the distal end of the five first-order veins. The corolla central vector was calculated as the vector connecting the three-tenths and the seven-tenths centroids (green points in **Figure 5A**) of the five first-order veins in the tube area. The vector was calculated from the proximal end of the corolla. The two traits were not affected by changes in image resolution or magnification (see **Supplement Data 1** for the MATLAB script).

## Results

### Volumetric and Surface Image Generation

Three-dimensional raw images of the 130 specimens were acquired. Some raw images are available on the Gigascience database (**Supplementary Table 1**; Hsu et al., 2020). The proposed procedures were applied to the raw images to generate volumetric and surface images. The volumetric images were on average 83.01% smaller in file size compared with the raw images.

### Detection of the First-Order Veins and Corolla Contours

**Figure 6:**
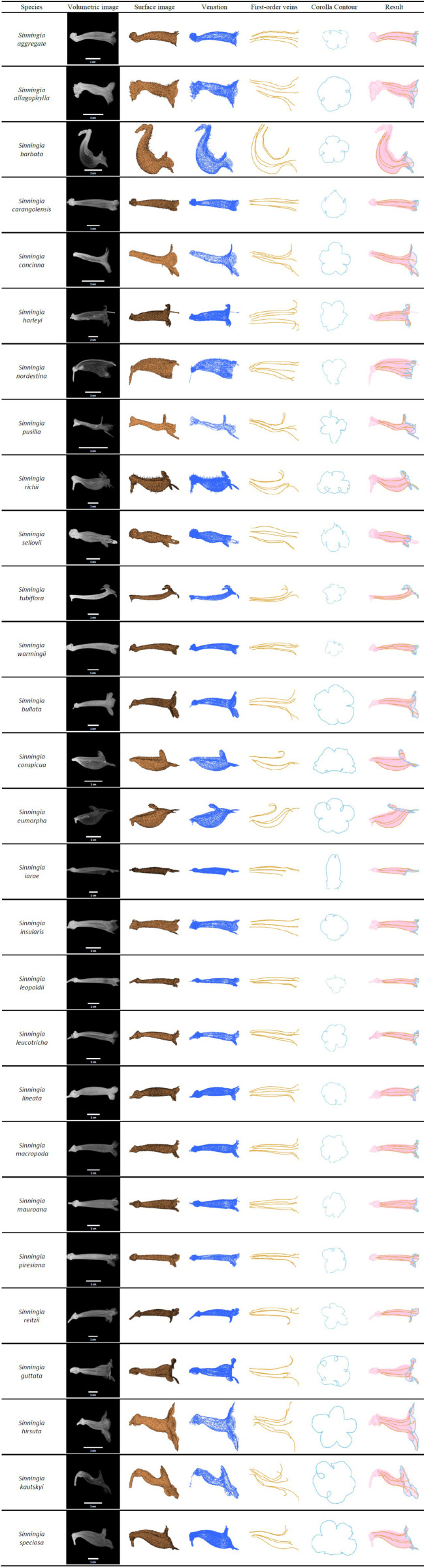
First-order veins and corolla contours of the species in subtribe Ligeriinae.

The algorithms were applied to the specimens to detect the first-order veins and corolla contours. **Figure 6** illustrates the volumetric image, surface image, venation, first-order veins, corolla contours, and the composition of the first-order veins and corolla contours for each species. The overall success rate for detecting both the first-order veins and corolla contours was 86.54% (**Table 1**, see **Supplementary Table 1** for detection results).

**Table 1:** Detection rates and pollination types for the species in subtribe Ligeriinae.

Species		Pollination type	Number of specimens	Detection rate (%)		Overall rate (%)
				First-order veins	Corolla contour	
Clade Corytholoma				85.67	90.87	81.71
	*S. aggregata*	Ornithophily	5	60.00	100.00	60.00
	*S. allagophylla*	Ornithophily	5	100.00	100.00	100.00
	*S. barbata*	Melittophily	5	100.00	100.00	100.00
	*S. carangolensis*	Ornithophily	6	100.00	83.33	83.33
	*S. concinna*	Melittophily	6	83.33	83.33	83.33
	*S. harleyi*	Ornithophily	5	100.00	100.00	100.00
	*S. nordestina*	Ornithophily	7	85.71	71.43	71.43
	*S. pusilla*	Melittophily	14	35.71	85.71	35.71
	*S. richii*	Melittophily	6	100.00	83.33	83.33
	*S. sellovii*	Ornithophily	6	83.33	83.33	83.33
	*S. tubiflora*	Phalaenophily	5	100.00	100.00	100.00
	*S. warmingii*	Ornithophily	5	80.00	100.00	80.00
Clade Dircaea				96.25	94.17	90.42
	*S. bullata*	Ornithophily	3	100.00	100.00	100.00
	*S. conspicua*	Melittophily	4	75.00	100.00	75.00
	*S. eumorpha*	Melittophily	4	100.00	75.00	75.00
	*S. iarae*	Ornithophily	3	100.00	100.00	100.00
	*S. insularis*	Ornithophily	3	100.00	100.00	100.00
	*S. leopoldii*	Ornithophily	3	100.00	100.00	100.00
	*S. leucotricha*	Ornithophily	3	100.00	100.00	100.00
	*S. lineata*	Ornithophily	5	80.00	80.00	60.00
	*S. macropoda*	Ornithophily	3	100.00	100.00	100.00
	*S. mauroana*	Ornithophily	4	100.00	75.00	75.00
	*S. piresiana*	Ornithophily	3	100.00	100.00	100.00
	*S. reitzii*	Ornithophily	3	100.00	100.00	100.00
Clade Sinningia				93.75	93.75	87.50
	*S. guttata*	Melittophily	4	75.00	100.00	75.00
	*S. hirsuta*	Melittophily	3	100.00	100.00	100.00
	*S. kautskyi*	Melittophily	4	75.00	75.00	75.00
	*S. speciosa*	Melittophily	3	100.00	100.00	100.00

### Morphological Traits

**Figure 7:**
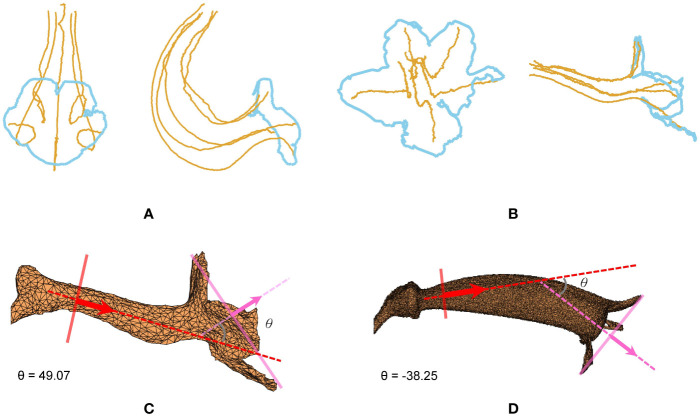
**(A)** First-order veins and corolla contour of *S. barbata*, **(B)** first-order veins and corolla contour of *S. pusilla*, **(C)** corolla angle of *S. pusilla* (*θ* = 49.07°), and **(D)** corolla angle of *S. reitzii* (*θ* = -38.25°). *S. barbata* (*ρ* = 0.200) and *S. pusilla* (*ρ* = 1.034), respectively, had the smallest and largest contour–vein ratios.

**Figure 8:**
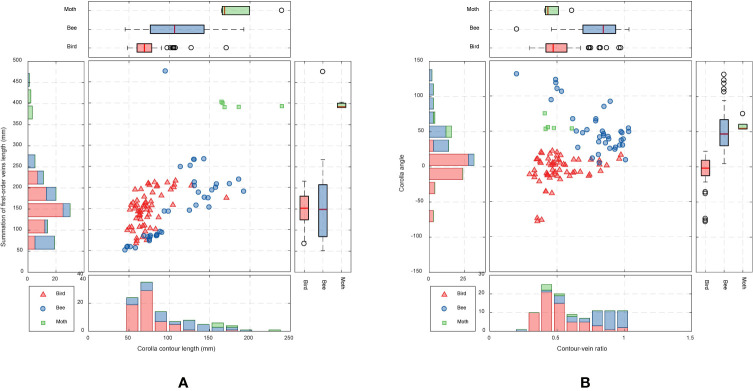
Scatter plots of **(A)** corolla contour length and length summation of first-order veins and **(B)** contour–vein ratio and corolla angle for the species in subtribe Ligeriinae. The traits are colored according to pollination types.

The contour–vein ratios and corolla angles were calculated for the 105 specimens in which the first-order veins and corolla contours were successfully detected. *S. pusilla* (**Figure 7B**) and *S. barbata* (**Figure 7A**), respectively, had the shortest (51.19 mm) and longest (475.02 mm) summation length of the first-order veins. *S. pusilla* (**Figure 7C**) and *S. tubiflora* (**Figure 7D**), respectively, had the shortest (45.38 mm) and longest (239.42 mm) corolla contours. Contour–vein ratios ranged between 0.200 and 1.034 (**Figure 8B**). *S. barbata* had a small contour–vein ratio (*ρ* = 0.200; **Figure 7A**), whereas *S. pusilla* had a large contour–vein ratio (*ρ* = 1.034; **Figure 7B**). Corolla angles ranged between -78.08° and 130.98°. *S. pusilla* bent upward (*θ* = 49.07°; **Figure 7C**), and *S.* *reitzii* bent downward (*θ* = -38.25°; **Figure 7D**).

Analyses were performed to examine if the defined morphological traits were associated with pollinator types. The pollination types of species were determined according to the study by Perret et al. (2003). Three pollination types were identified (**Table 1**): ornithophilic (bird-pollinated), melittophilic (bee-pollinated), and phalaenophilic (moth-pollinated). We observed that the ornithophilic species developed corollas with straight and narrow tubes. The mean corolla contour length, mean absolute corolla angle, and mean contour–vein ratio of the ornithophilic species were 73.55 mm, 14.63°, and 0.52, respectively. The three traits of the ornithophilic species were significantly smaller than those of the other species (**Figure 8**; *P* < 5.35^-10^). These traits reflect the behaviors of hummingbirds, which are plant pollination mediators. When hummingbirds visit flowers, they place their beaks into corolla tubes to acquire nectar (Serrano-Serrano et al., 2017). Therefore, ornithophilic species developed corollas with narrow and straight tubes without broad lobes. We also observed that the melittophilic species developed corollas with lobes that bend upward (*θ* ≥ 4.62°) and tubes that dilate. The mean corolla contour length, mean corolla angle, and mean contour–vein ratio of the melittophilic species were 110.21 mm, 52.99°, and 0.80, respectively. These three traits of the melittophilic species were significantly different to those of the ornithophilic species (**Figure 7**; *P <*1.92^-12^). These traits are related to the behavior of bees. Bees usually land on ventral petals and reach the base of corollas to acquire nectar (SanMartin-Gajardo and Sazima, 2004). Therefore, the ornithophilic species developed corollas that bend upward with dilated tubes.

## Discussion

### Failure Case Examination

**Figure 9:**
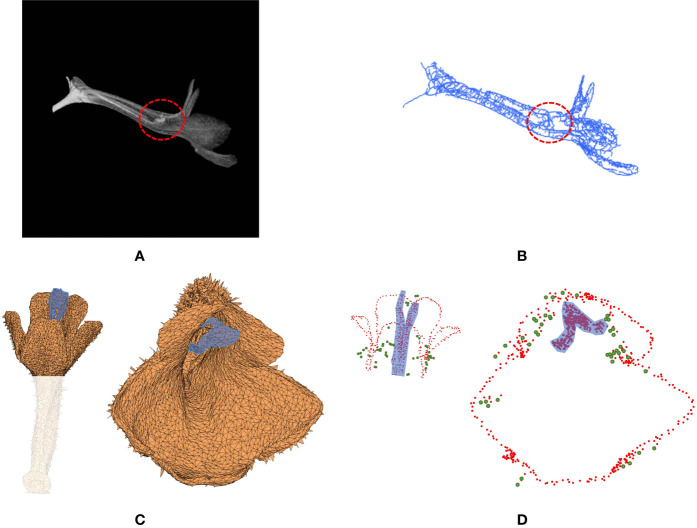
**(A)** Volumetric image and **(B)** venation of *S. pusilla*, and **(C)** surface image and **(D)** contour nodes of *S. nordestina*. In **(A, B)**, the red circle indicates the area where the pistil and stamens touched the corolla tube. In **(C, D)**, the region highlighted in blue indicates the pistil and the stamen protruding from the corolla tube.

The failure cases in detecting venation or corolla contour were examined. False detection mostly occurred at the pistils, stamens, or pubescence of the corollas. Certain species (e.g., *S. pusilla* and *S. aggregata*) had pistils or stamens that touched the tube of the corollas (red circle in **Figure 9A**). Parts of pistils and stamens were falsely recognized as venation points (**Figure 9B**). Some other species (e.g., *S. nordestina* and *S. sellovii*) had pistils or stamens longer than the corolla tube (blue area in **Figure 9C**). Parts of the pistils and stamens were falsely recognized as contour nodes (red points in the blue area in **Figure 9D**). Some other species (e.g., *S. nordestina* and *S. richii*) had pubescence on the petal surface (**Figure 9C**). The tips of trichomes were falsely recognized as contour nodes (green nodes in **Figure 9D**).

### Applicability to Tissues Fixed in an Alcohol Solution

**Figure 10:**
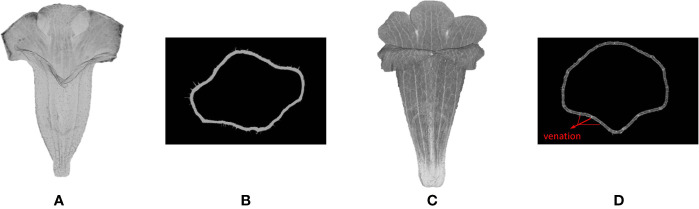
**(A)** Volumetric and **(B)** slice image of corolla (*S. conspicua*) fixed in an alcohol solution, and **(C)** volumetric and **(D)** slice image of corolla (*S. conspicua*) reserved in fresh. The venation of the fresh corolla is observed.

The proposed procedure was applied to 3D images of floral specimens preserved in an alcohol solution (70% ethanol with 2% glycerol). This approach failed to identify the venation of the specimens. Once the corollas were fixed in an alcohol solution, all the tissues were infiltrated by the liquid (**Figure 10A**). The contrast between the venation and the mesophyll tissue in the volumetric images (**Figure 10B**) was too low compared with the image in fresh (**Figures 10C, D**), and thus venation could not be detected using Hessian of Gaussian. However, corolla contours of specimens fixed in an alcohol solution were successfully detected.

### Applicability to Other Species

**Figure 11:**
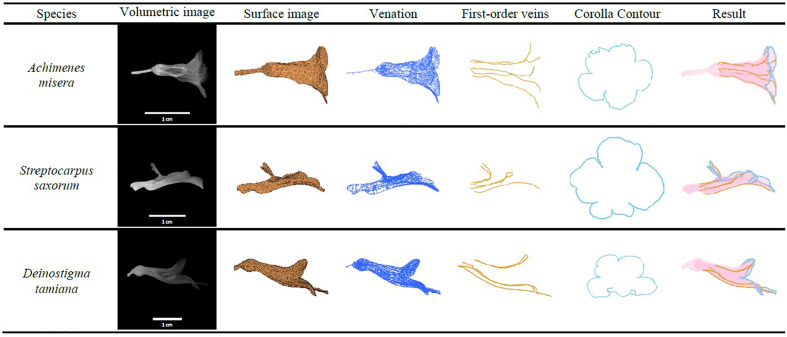
First-order veins and corolla contours of *A. misera*, *S. saxorum*, and *D. tamiana*.

The proposed approach was applied to the corollas of the species that do not belong to the subtribe Ligeriinae. The species investigated were *Achimenes misera* in subtribe Gloxiniinae, *Streptocarpus saxorum* in subtribe Streptocarpinae, and *Deinostigma tamiana* in subtribe Didymocarpinae. Both the first-order veins and corolla contours of the specimens were successfully detected (**Figure 11**). The results indicated that the proposed approach could be generalized to fresh flower specimens other than the species of subtribe Ligeriinae.

### Potential Implications to Other Subjects

The proposed method has the potential to be applied to identifying the traits of other botanic subjects. The detection of the first-order veins uses the grayscale differences between the vein and neighboring tissues; the detection of the corolla contours uses the separation between the corolla and the background. The grayscale differences between tissues and the separation between study objects and background exist in 3D CT images of other botanic subjects, such as woods (Brodersen and Roddy, 2016), roots (Mairhofer et al., 2015), and plant fossils (Gee, 2013). With appropriate modifications, the developed algorithms in this study may contribute the researchers in these subjects to automate the trait quantification and may shed light on exploring the mystery of plant morphologies in ecology and evolutionary biology.

## Concluding Remarks

This study proposed an approach for semiautomatically identifying the first-order veins of corollas and automatically identifying contours of corollas in 3D images. 3D images of 130 corolla specimens in the subtribe Ligeriinae were collected using a micro-CT scanner. The raw images were converted to volumetric images and surface images. First-order veins of the corollas were then identified from the volumetric image using Hessian of Gaussian and Dijkstra’s algorithm. Corolla contours were identified from the surface image using vector harmony and node distance thresholding. The overall success rate for detecting both the first-order veins and corolla contours was 86.54%. Two traits, contour–vein ratio and corolla angle, were defined and quantified using the first-order vein and corolla contour results to reveal the relationship between corolla shapes and pollination types. Contour–vein ratios of the corollas ranged from 0.200 to 1.034, and corolla angles ranged from -78.08 to 130.98°. The mean corolla contour length, mean absolute corolla angle, and mean contour–vein ratio of the ornithophilic species were significantly smaller than those of the other species. The mean corolla contour length, mean corolla angle, and mean contour–vein ratio of the melittophilic species were significantly larger than those of the ornithophilic species. Tests also demonstrated that the proposed method could be used to identify first-order veins and corolla contours in certain species in the subtribes Gloxiniinae, Streptocarpinae, and Didymocarpinae.

## Data Availability Statement

Publicly available datasets were analyzed in this study. This data can be found here: (http://gigadb.org/dataset/100681).

## Author Contributions

Y-FK was responsible for the experiment design. H-CH prepared flower materials. Y-HW, W-CC, and C-HL performed the program development. Y-HW, H-CH, and Y-FK analyzed the data, and were responsible for writing the manuscript. H-CH and Y-FK contributed to reviewing the manuscript.

## Funding

This work was supported by the National Science Council (Ministry of Science and Technology) of Taiwan grant NSC-101-2313-B-002-050-MY3.

## Conflict of Interest

The authors declare that the research was conducted in the absence of any commercial or financial relationships that could be construed as a potential conflict of interest.

## Supplementary Material

Supplementary Data 1 MATLAB script for the volumetric image generation and surface image generation, the identification of first-order veins, the identification of corolla contours, and the morphological trait quantification.
