# Supplementary material for: Automatic Identification of First-Order Veins and Corolla Contours in Three-Dimensional Floral Images
Source: Front Plant Sci. 2020 Sep 16;11:549699. doi: 10.3389/fpls.2020.549699 (PMC7525071; doi:10.3389/fpls.2020.549699)
Supplement: Supplementary file 2 [file Table_1.docx]

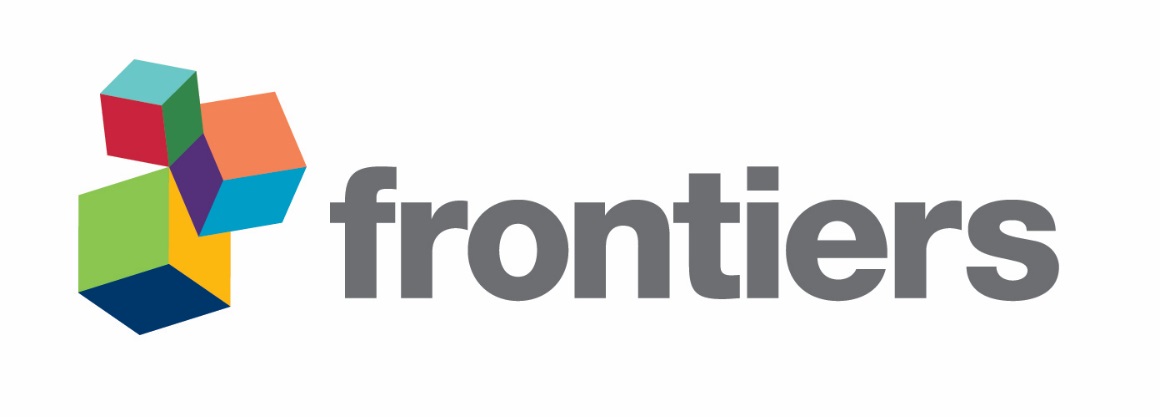


Supplementary Material

**Supplementary Table 1:** Data of 130 specimens from 28 species in subtribe Ligeriieae.

| **Species** | | **Image volume (voxels)** | | **Detection result** | | **GigaDB**  **sample ID** |
| --- | --- | --- | --- | --- | --- | --- |
|  |  | **Original** | **Processed** | **First-order veins** | **Corolla contour** |  |
| **Clade Corytholoma** | |  |  |  |  |  |
|  | *Sinningia aggregate* | 1000×1000×980 | 243×281×969 | v | v | K039092_01 |
|  |  | 1000×1000×890 | 274×323×872 | v | v | K039092_02 |
|  |  | 1000×1000×910 | 287×274×892 | v | v | K039092_03 |
|  |  | 1000×1000×810 | 240×237×787 | -- | v | K039092_04 |
|  |  | 1000×1000×810 | 252×238×792 | -- | v | K039092_05 |
|  | *Sinningia allagophylla* | 1000×1000×570 | 313×327×568 | v | v | K039099_01 |
|  |  | 1000×1000×610 | 243×374×589 | v | v | K039099_02 |
|  |  | 1000×1000×540 | 328×311×527 | v | v | K039099_03 |
|  |  | 1000×1000×550 | 367×339×537 | v | v | K039099_04 |
|  |  | 1000×1000×540 | 398×373×509 | v | v | K039099_05 |
|  | *Sinningia barbata* | 984×984×620 | 709×328×617 | v | v | HC1206-a_09 |
|  |  | 984×984×700 | 463×347×694 | v | v | HC1206-d_10 |
|  |  | 984×984×655 | 512×344×644 | v | v | K039105_02 |
|  |  | 984×984×650 | 505×424×639 | v | v | K039105_03 |
|  |  | 984×984×635 | 457×293×622 | v | v | K039105_05 |
|  | *Sinningia carangolensis* | 1000×1000×1140 | 301×293×1129 | v | v | HC1912-2_02 |
|  |  | 1000×1000×1090 | 269×274×1085 | v | -- | HC1912-2_03 |
|  |  | 1000×1000×1050 | 263×344×1046 | v | v | HC1912-b_01 |
|  |  | 1000×1000×1170 | 276×309×1140 | v | v | K039112_02 |
|  |  | 1000×1000×1220 | 251×306×1204 | v | v | K039112_03 |
|  |  | 1000×1000×1150 | 292×286×1124 | v | v | K039112_04 |
|  | *Sinningia concinna* | 1000×1000×505 | 328×332×499 | v | v | K039117_01 |
|  |  | 1000×1000×629 | 411×448×624 | -- | -- | -- |
|  |  | 1000×1000×553 | 450×477×547 | v | v | HC2202-t_05 |
|  |  | 1000×1000×610 | 410×387×597 | v | v | -- |
|  |  | 1000×1000×510 | 353×365×510 | v | v | HC2202-t_04 |
|  |  | 1000×1000×580 | 423×461×572 | v | v | -- |
|  | *Sinningia harleyi* | 1000×1000×1400 | 581×652×1398 | v | v | K039135_01 |
|  |  | 1000×1000×1280 | 650×669×1268 | v | v | K039135_03 |
|  |  | 1000×1000×1320 | 611×692×1312 | v | v | K039135_05 |
|  |  | 1000×1000×1230 | 666×618×1214 | v | v | HC3403-8_09 |
|  |  | 1000×1000×1340 | 661×552×1335 | v | v | HC3403-8_11 |
|  | *Sinningia nordestina* | 1000×1000×780 | 490×378×762 | -- | -- | K039168_01 |
|  |  | 1000×1000×780 | 437×297×779 | v | v | K039168_02 |
|  |  | 1000×1000×800 | 325×343×786 | v | v | K039168_03 |
|  |  | 1000×1000×780 | 340×315×769 | v | v | K039168_05 |
|  |  | 1000×1000×780 | 445×313×774 | v | v | HC5504-1_03 |
|  |  | 1000×1000×780 | 498×344×755 | v | v | HC5504-3_03 |
|  |  | 1000×1000×750 | 342×337×721 | v | -- | HC5504-3_04 |
|  | *Sinningia pusilla* | 1000×1000×423 | 226×286×419 | v | v | -- |
|  |  | 1000×1000×418 | 217×265×414 | v | v | -- |
|  |  | 1000×1000×446 | 229×235×439 | v | v | K039170_01 |
|  |  | 1000×1000×401 | 193×208×397 | -- | v | K039170_02 |
|  |  | 1000×1000×385 | 175×211×380 | -- | v | K039170_03 |
|  |  | 1000×1000×392 | 220×255×382 | -- | v | K039170_04 |
|  |  | 1000×1000×386 | 254×242×384 | v | v | K039170_05 |
|  |  | 1000×1000×387 | 265×293×384 | -- | v | HC5803-7_09 |
|  |  | 1000×1000×376 | 175×165×372 | -- | v | HC5803-2_01 |
|  |  | 1000×1000×420 | 255×249×414 | -- | v | -- |
|  |  | 1000×1000×390 | 159×154×382 | -- | v | -- |
|  |  | 1000×1000×377 | 286×246×374 | -- | -- | K039171_01 |
|  |  | 1000×1000×469 | 322×297×466 | v | v | K039172_01 |
|  |  | 1000×1000×580 | 244×284×568 | -- | -- | -- |
|  | *Sinningia richii* | 1000×1000×1150 | 667×544×1131 | v | v | K039174_02 |
|  |  | 1000×1000×1160 | 565×604×1130 | v | v | K039174_03 |
|  |  | 1000×1000×1060 | 451×535×1013 | v | v | K039174_04 |
|  |  | 1000×1000×1060 | 625×560×1045 | v | v | K039175_01 |
|  |  | 1000×1000×1060 | 535×483×1029 | v | v | K039174_05 |
|  |  | 1000×1000×1060 | 663×556×1034 | v | -- | K039176_01 |
|  | *Sinningia sellovii* | 1000×1000×940 | 258×356×937 | v | v | K039184_03 |
|  |  | 1000×1000×1000 | 265×324×999 | v | v | K039184_04 |
|  |  | 1000×1000×930 | 274×304×923 | v | v | K039184_05 |
|  |  | 1000×1000×800 | 215×283×796 | -- | -- | K039186_11 |
|  |  | 1000×1000×1000 | 258×307×994 | v | v | K039186_12 |
|  |  | 1000×1000×930 | 257×252×920 | v | v | K039186_13 |
|  | *Sinningia tubiflora* | 984×984×1235 | 486×426×1230 | v | v | K039197_02 |
|  |  | 984×984×1230 | 400×367×1224 | v | v | K039199_02 |
|  |  | 984×984×1220 | 417×517×1212 | v | v | K039200_01 |
|  |  | 984×984×1195 | 414×395×1187 | v | v | K039200_02 |
|  |  | 984×984×1165 | 464×490×1160 | v | v | K039200_03 |
|  | *Sinningia warmingii* | 1000×1000×1170 | 285×321×1139 | -- | v | K039205_04 |
|  |  | 1000×1000×1350 | 318×323×1332 | v | v | K039209_10 |
|  |  | 1000×1000×1380 | 327×325×1365 | v | v | K039209_11 |
|  |  | 1000×1000×1420 | 314×324×1407 | v | v | -- |
|  |  | 1000×1000×1420 | 393×364×1401 | v | v | -- |
| **Clade Dircaea** | |  |  |  |  |  |
|  | *Sinningia bullata* | 1000×1000×1100 | 603×597×1093 | v | v | -- |
|  |  | 1000×1000×1290 | 638×665×1281 | v | v | -- |
|  |  | 1000×1000×1230 | 561×618×1216 | v | v | -- |
|  | *Sinningia conspicua* | 1000×1000×1440 | 899×695×1426 | -- | v | -- |
|  |  | 984×984×675 | 404×320×667 | v | v | -- |
|  |  | 984×984×710 | 411×311×697 | v | v | -- |
|  |  | 984×984×685 | 434×326×679 | v | v | -- |
|  | *Sinningia eumorpha* | 984×984×683 | 515×374×680 | v | -- | -- |
|  |  | 984×984×744 | 539×354×739 | v | v | -- |
|  |  | 984×984×687 | 897×426×685 | v | v | -- |
|  |  | 984×984×655 | 646×331×653 | v | v | -- |
|  | *Sinningia iarae* | 1000×1000×1800 | 314×259×1769 | v | v | -- |
|  |  | 1000×1000×1800 | 317×263×1770 | v | v | -- |
|  |  | 1000×1000×1800 | 293×335×1789 | v | v | -- |
|  | *Sinningia insularis* | 1000×1000×950 | 313×292×939 | v | v | -- |
|  |  | 1000×1000×930 | 328×331×926 | v | v | -- |
|  |  | 1000×1000×910 | 295×289×907 | v | v | -- |
|  | *Sinningia leopoldii* | 1000×1000×1200 | 281×250×1180 | v | v | -- |
|  |  | 1000×1000×1310 | 252×303×1301 | v | v | -- |
|  |  | 1000×1000×1100 | 298×280×1093 | v | v | -- |
|  | *Sinningia leucotricha* | 1000×1000×1030 | 314×334×1030 | v | v | -- |
|  |  | 1000×1000×1080 | 322×309×1062 | v | v | -- |
|  |  | 1000×1000×1010 | 390×373×1003 | v | v | -- |
|  | *Sinningia lineata* | 1000×1000×1150 | 342×378×1142 | v | v | -- |
|  |  | 1000×1000×1130 | 406×402×1120 | v | v | -- |
|  |  | 1000×1000×1150 | 407×388×1139 | v | -- | -- |
|  |  | 1000×1000×1140 | 390×493×1113 | -- | v | -- |
|  |  | 1000×1000×1130 | 428×351×1107 | v | v | -- |
|  | *Sinningia macropoda* | 1000×1000×1120 | 409×412×1111 | v | v | -- |
|  |  | 1000×1000×1070 | 376×369×1067 | v | v | -- |
|  |  | 1000×1000×1010 | 304×322×1002 | v | v | -- |
|  | *Sinningia mauroana* | 1000×1000×1140 | 316×348×1121 | v | v | -- |
|  |  | 1000×1000×1150 | 304×342×1103 | v | -- | -- |
|  |  | 1000×1000×1180 | 319×370×1161 | v | v | -- |
|  |  | 1000×1000×950 | 249×278×927 | v | v | -- |
|  | *Sinningia piresiana* | 1000×1000×990 | 272×281×977 | v | v | -- |
|  |  | 1000×1000×960 | 290×282×955 | v | v | -- |
|  |  | 1000×1000×970 | 297×310×960 | v | v | -- |
|  | *Sinningia reitzii* | 1000×1000×1550 | 515×462×1540 | v | v | -- |
|  |  | 1000×1000×1480 | 486×498×1472 | v | v | -- |
|  |  | 1000×1000×1440 | 431×445×1428 | v | v | -- |
| **Clade Sinningia** | |  |  |  |  |  |
|  | *Sinningia guttata* | 1000×1000×1340 | 694×650×1333 | v | v | -- |
|  |  | 1000×1000×1380 | 652×751×1377 | v | v | -- |
|  |  | 1000×1000×1400 | 577×712×1395 | -- | v | -- |
|  |  | 1000×1000×1260 | 673×607×1253 | v | v | -- |
|  | *Sinningia hirsuta* | 1000×1000×500 | 480×447×490 | v | v | -- |
|  |  | 1000×1000×580 | 575×479×565 | v | v | -- |
|  |  | 1000×1000×650 | 478×469×630 | v | v | -- |
|  | *Sinningia kautskyi* | 1000×1000×580 | 379×376×565 | v | v | -- |
|  |  | 1000×1000×700 | 466×260×660 | v | v | -- |
|  |  | 1000×1000×770 | 441×349×726 | -- | -- | -- |
|  |  | 1000×1000×590 | 316×414×561 | v | v | -- |
|  | *Sinningia speciosa* | 1000×1000×1170 | 743×577×1168 | v | v | -- |
|  |  | 1000×1000×1410 | 754×734×1397 | v | v | -- |
|  |  | 1000×1000×1360 | 697×704×1345 | v | v | -- |
